# OntoFox: web-based support for ontology reuse

**DOI:** 10.1186/1756-0500-3-175

**Published:** 2010-06-22

**Authors:** Zuoshuang Xiang, Mélanie Courtot, Ryan R Brinkman, Alan Ruttenberg, Yongqun He

**Affiliations:** 1 Unit for Laboratory Animal Medicine, University of Michigan Medical School, Ann Arbor, MI 48109, USA; 2 Department of Microbiology and Immunology, University of Michigan Medical School, Ann Arbor, MI 48109, USA; 3 Center for Computational Medicine and Bioinformatics, University of Michigan Medical School, Ann Arbor, MI 48109, USA; 4 Terry Fox Laboratory, BC Cancer Agency, 675 West 10th Avenue, Vancouver, BC, Canada; 5 Department of Medical Genetics, University of British Columbia, Vancouver, BC, Canada; 6 Science Commons, 77 Massachusetts Avenue Room E25-131, Cambridge MA 02139, USA

## Abstract

**Background:**

Ontology development is a rapidly growing area of research, especially in the life sciences domain. To promote collaboration and interoperability between different projects, the OBO Foundry principles require that these ontologies be open and non-redundant, avoiding duplication of terms through the re-use of existing resources. As current options to do so present various difficulties, a new approach, MIREOT, allows specifying import of single terms. Initial implementations allow for controlled import of selected annotations and certain classes of related terms.

**Findings:**

OntoFox http://ontofox.hegroup.org/ is a web-based system that allows users to input terms, fetch selected properties, annotations, and certain classes of related terms from the source ontologies and save the results using the RDF/XML serialization of the Web Ontology Language (OWL). Compared to an initial implementation of MIREOT, OntoFox allows additional and more easily configurable options for selecting and rewriting annotation properties, and for inclusion of all or a computed subset of terms between low and top level terms. Additional methods for including related classes include a SPARQL-based ontology term retrieval algorithm that extracts terms related to a given set of signature terms and an option to extract the hierarchy rooted at a specified ontology term. OntoFox's output can be directly imported into a developer's ontology. OntoFox currently supports term retrieval from a selection of 15 ontologies accessible via SPARQL endpoints and allows users to extend this by specifying additional endpoints. An OntoFox application in the development of the Vaccine Ontology (VO) is demonstrated.

**Conclusions:**

OntoFox provides a timely publicly available service, providing different options for users to collect terms from external ontologies, making them available for reuse by import into client OWL ontologies.

## Background

Biomedical ontologies are sets of terms and relations that represent entities in the scientific world and how they relate to each other. Terms are associated with documentation and definitions, which are, ideally, expressed in formal logic in order to support automated reasoning [1-3]. Ontologies have dramatically changed how biomedical research is conducted. For example, since the Gene Ontology (GO) was first published in 2000 [1], it has been used and cited in more than 2000 peer-reviewed journal articles [4]. Ontologies have been used in various applications, such as gene expression data analysis [1], literature mining [5], and as the underpinning of a semantic web [6]. There are currently more than 150 biomedical ontologies and 700,000 entities in the NCBO BioPortal http://bioportal.bioontology.org/. With new resources continuously being developed, maximizing ontology sharing and interoperability has become a growing concern [7,8].

The development of a new biomedical ontology covering a specific domain is often an ambitious, time-consuming project, usually requiring extensive cross-community collaboration. The OBO Foundry is an open community that has established a set of principles for ontology development with the goal of creating a suite of interoperable reference ontologies in the biomedical domain [3]. These principles require that member ontologies be open, orthogonal, expressed in a common shared syntax, and designed to possess a common space of identifiers. One way of meeting the goal of interoperability is to reuse existing resources by importing them into the to-be-created ontology. For example, the Vaccine Ontology (VO, http://www.violinet.org/vaccineontology) [9] relies on many terms (*e.g*., administering substance *in vivo*) already described by other biomedical ontologies, such as the Ontology for Biomedical Investigations (OBI; http://purl.obolibrary.org/obo/obi).

OWL currently only provides a mechanism to import ontologies as a whole [10]. This approach is reasonable and recommended for small ontologies that are designed in ways consistent with the importing ontology. However, in many cases this is neither practical nor needed. For example, the source ontology may be too large for editing tools, use different design patterns, or be at an early stage of development. Nevertheless, individual terms in such ontologies may be well-defined and therefore desirable to reuse. As an example, the Chemical Entities of Biological Interest ontology (ChEBI; http://www.ebi.ac.uk/chebi/) currently includes over 455,000 terms. Importing CHEBI as a whole into a target ontology is impractical given current editing (e.g., Protégé ontology editor [11]) and reasoning tools (*e.g*., Pellet [12] and Fact ++ [13]). Protégé can perhaps handle in the low 10,000s of terms before becoming too slow to use, and with the addition of complex logical restrictions, reasoning performance is non-interactive with the resources used.

As a practical alternative to importing whole ontologies, MIREOT (Minimum Information to Reference an External Ontology Term) was developed in the context of the OBI project [14]. MIREOT proposes selective use of classes from external ontologies that are of direct interest to a target ontology, instead of importing external ontologies as a whole. For example, both the OBI and the VO require the ontology term '*homo sapiens*', and have decided to use the NCBI Taxonomy Ontology (NCBITaxon) as a common resource for naming taxonomic groups. The corresponding URI for '*homo sapiens*' is http://purl.org/obo/owl/NCBITaxon#NCBITaxon_9606. MIREOT specifies that the minimal information needed to specify reuse of this term is (i) this URI, (ii) the URI of the parent term in the importing ontology (http://purl.obolibrary.org/obo/OBI_0100026, *organism*), and (iii) the ontology IRI of the source ontology. Based on this minimal information, an automated process can be used to retrieve (and periodically refresh) chosen additional information such as the preferred label for the term and elements of the taxonomic hierarchy. MIREOT is being used in a number of ontology projects, for example, OBI, VO, the Influenza Ontology (InfluenzO; http://sourceforge.net/projects/influenzo/), Neural ElectroMagnetic Ontologies (NEMO; http://nemo.nic.uoregon.edu/wiki/NEMO), ontologies developed in the Neuroscience Information Framework (NIF; https://confluence.crbs.ucsd.edu/display/NIF/), and as part of the eagle-i project https://www.eagle-i.org/home/.

While editing tools commonly provide means to reference an external term by directly setting its URI, one must also manually enter auxiliary information necessary for practical editing, such as the label and definition, and update such information if the source ontology changes. In addition, it is often desirable to import additional related terms. For example, when the Vaccine Ontology imports a species term, the inclusion of some of its superclasses allows for queries at different taxonomic ranks *(e.g*., kingdom, phylum, and species). To address these issues, an initial implementation based on MIREOT was created to facilitate managing the tedious aspects of this process automatically http://obi-ontology.org/page/MIREOT.

The developers of the MIREOT guideline recognize that such an approach is a balanced compromise. Importing only selected information means that incomplete or incorrect inferences could conceivably be made. Technical approaches such as module extraction [12,15-17] promise to preserve correct inference, under a variety of assumptions, by computationally selecting portions of an ontology. Recent work on modularization casts it as a process that fragments existing ontologies into a set of smaller and possibly interconnected parts or modules [12,15-18] that can then be reused as units of ontology [19].

There have been several approaches to computing modules [20]. Structural approaches use the syntax of the axioms of ontologies and mostly only consider the induced *is-a *hierarchy [17,21]. Logic-based approaches take into account the consequences of ontologies and require that this extracted module captures the meaning of the imported terms used, *i.e*., includes all axioms relevant to the meaning of these terms. However, Grau et al. [22] proved that it is undecidable, even for description logics simpler than OWL-DL, to determine whether a subset of an ontology is a minimal logic-based module.

These approaches are relatively new, experience using them is limited, and our experience with current Web-based implementations has found them to be unreliable. Moreover the methods do not provide ways to avoid import of certain terms or axioms that might not be considered desirable, or have other issues that prevent their easy use [23]. Nonetheless the syntactic locality approach these methods use is applicable to single-term import and so is compatible with the MIREOT approach.

The OBI project has an implementation of the MIREOT mechanism that demonstrates the feasibility of the approach. It is, however, command line-based and requires the specification of terms either by command-line scripts or construction of an ontology document. Specification of which ancillary information should be incorporated is by writing SPARQL queries [24], restricting its adoption by less technically able users. To facilitate application of the MIREOT guideline by the wider ontology community a more user-friendly system facilitating the import and update of external terms into a target ontology is desired. In addition, while MIREOT provides a practical yet simple approach to specifying external ontology terms, the OBI implementation does not provide the ability to consider restrictions on imported terms that a user may desire to import. To preserve the meaning of the imported terms, ontology developers might like to use ontology module extraction, *e.g*., extraction of the target class and its transitively related (via restriction) closure [17]. Ontology developers may also want the flexibility of including no superclasses, only one direct superclass, all superclasses to the top class, or a subset of all superclasses for a term, in order to provide additional relevant domain terms for their users.

To address these needs for ontology reuse, we have developed OntoFox http://ontofox.hegroup.org/, a web-based application implementing the MIREOT and related ontology term extraction strategies. OntoFox facilitates ontology development by automatically fetching properties, annotations, and related terms from external ontology terms and saving the results as OWL serialized as RDF/XML [25] suitable for use with the OWL import directive. OntoFox provides a web-based package of solutions for ontology developers to extract, for subsequent import, different sets of ontology terms by following and expanding the initial MIREOT implementation and by developing related ontology term extraction methods based on SPARQL [26]. In this manuscript, we will introduce the general OntoFox web system, describe the ways it lets users describe which properties and related terms should be imported, and demonstrate how OntoFox is used in the VO development.

## Methods

### OntoFox system architecture

**Figure 1:**
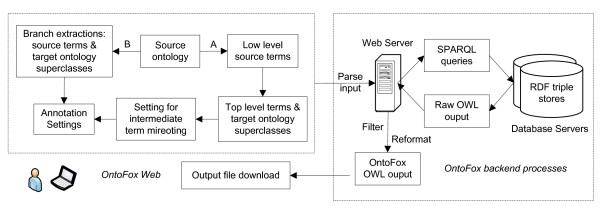
**OntoFox workflow**. The input data is parsed internally by the OntoFox web server. SPARQL queries are then constructed and used to query remote RDF triple stores, containing the RDF triples of source ontologies. After successful query execution, an OWL output file is generated and provided to the user for download.

OntoFox uses a simple text format and web forms for data input in a user-friendly implementation, and is designed to not require any programming skills. OntoFox is implemented using a three-tier system architecture. At the front-end, data can be submitted using either web forms or by uploading a plain text input file. The input data are then processed using PHP and Java., and SPARQL (middle-tier, application server) queries are then executed against an RDF triple store (back-end, database server), currently the Neurocommons SPARQL endpoint [6]. The web server then processes the result of each SPARQL query sent by the back-end server; as a result an RDF/XML file is created and offered for download to the user (Figure 1).

As OntoFox is a web-based system, it is accessible everywhere through the Internet without need for additional software installation. The techniques used in the OntoFox web application were chosen for maximum compatibility by using established W3C standards, specifically, OWL as a web ontology language, RDF/XML as its serialization, and SPARQL for queries.

### OntoFox three-tier structure implementation

#### (1) OntoFox web interface

The OntoFox web interface is designed based on iterative testing, thus far informal usability testing and feedback from initial users, following a spiral software development model [27]. It accepts the input from the user, via either web forms or uploading of a local text file, and presents the output data after query processing.

**Figure 2:**
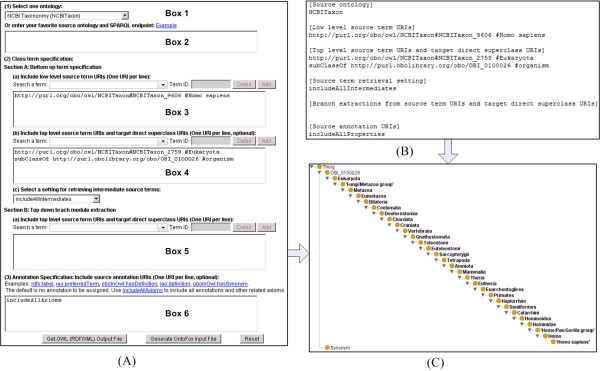
**OntoFox retrieval of the term '*homo sapiens*' from the NCBI Taxonomy Ontology (NCBITaxon)**. Input data can be entered via web-based forms (A) or text file upload (B). The output OWL file [Additional file 1] can be visualized using Protégé (C). All terms from '*homo sapiens*' up to Eukaryota are retrieved. Synonyms used to annotate each term are also included.

Finding and entering the URIs for desired terms can be tedious. To speed up the term specification process, we implemented an ontology term suggestion feature based on auto-completion of the string of text entered by users after selecting the desired source ontology. The OntoFox server offers a list of potential matches, and upon selection, the associated term ID will show up in an input box next to the label. Additionally, the "Detail" hyperlink next to the term ID provides easy access to an interactive ontology browser allowing visual confirmation of the term definition and its position in the hierarchical ontology tree structure. Lastly, as shown in Figure 2, the user can click "Add" next to "Detail" to insert the full URL of the selected term into the input textbox on the web interface.

#### (2) Data processing by the web application

The OntoFox application server runs on a Dell PowerEdge 2580 server running the Red Hat Linux operating system (Red Hat Enterprise Linux 5 server). PHP and Java are used as programming languages in the web application server. General web-based programming and query submission are written using PHP. The OWL API http://owlapi.sourceforge.net/, a Java API for manipulating OWL files, is used in OntoFox to read, process, and rewrite OWL files and save the final results as one OWL file after merging individual query results.

#### (3) Data storage and access

The OntoFox internal RDF database server runs on a separate Dell PowerEdge 2580 server. The database server is powered by the OpenLink Virtuoso database engine http://www.openlinksw.com/virtuoso/. While VO is loaded within this Virtuoso server, OntoFox also uses RDF data stored in other web accessible servers, for example, the Neurocommons knowledge management platform [6]. Fifteen biomedical ontologies generally used within the OBO community are available for users to select as source ontologies within OntoFox (Table 1). These ontologies, initially chosen to support VO development, were selected based on their specificity, community support, and maturity. They all adhere to a strict deprecation policy, ensuring that the meaning of each term remains stable until the term is deprecated. Though nothing bars serving more resources, these 15 ontologies were all that were required to cover all information needed for import via MIREOT during the VO development. Users can choose to provide another source ontology URI and corresponding SPARQL endpoint, allowing retrieval of terms outside of the OntoFox source ontology repository resources; however this is done at their own risk as term stability is not guaranteed.

**Table 1:** The 15 source ontologies currently available in OntoFox

Ontology	Source Ontology URI	Example of source Ontology Term URI
CARO	http://purl.org/obo/owl/CARO	http://purl.org/obo/owl/CARO#CARO_0000040
CHEBI	http://purl.org/obo/owl/CHEBI	http://purl.org/obo/owl/CHEBI#CHEBI_48999
CL	http://purl.org/obo/owl/CL	http://purl.org/obo/owl/CL#CL_0000799
DOID	http://purl.org/obo/owl/DOID	http://purl.org/obo/owl/DOID#DOID_12685
ENVO	http://purl.org/obo/owl/ENVO	http://purl.org/obo/owl/ENVO#ENVO_00000483
FMA	http://purl.org/obo/owl/FMA	http://purl.org/obo/owl/FMA#FMA_9712
GO	http://purl.org/obo/owl/GO	http://purl.org/obo/owl/GO#GO_0043152
IDO	http://purl.obolibrary.org/obo/ido.owl	http://purl.obolibrary.org/obo/IDO_0000064
MP	http://purl.org/obo/owl/MP	http://purl.org/obo/owl/MP#MP_0000026
NCBITaxon	http://purl.org/obo/owl/NCBITaxon	http://purl.org/obo/owl/NCBITaxon#NCBITaxon_263
OBI	http://purl.obolibrary.org/obo/obi.owl	http://purl.obolibrary.org/obo/OBI_0100026
PATO	http://purl.org/obo/owl/PATO	http://purl.org/obo/owl/PATO#PATO_0001793
PRO	http://purl.org/obo/owl/PRO	http://purl.org/obo/owl/PRO#PRO_000001795
SO	http://purl.org/obo/owl/SO	http://purl.org/obo/owl/SO#SO_0001288
VO	http://purl.obolibrary.org/obo/vo.owl	http://purl.obolibrary.org/obo/VO_0000001

### Evaluation of OntoFox SPARQL retrieval of related terms

To compare the performance of the OntoFox SPARQL related term retrieval approach with the OWLAPI modularization, three sets of signature data were used. The first two sets of signature data include either one term (e.g., the OBI term 'antigen') or a list of OBI terms that were imported to VO. The third set of signature terms for modularization includes all terms in the Neuroscience Information Framework (NIF) Lexicon ontology (nif.owl; http://ontology.neuinfo.org/NIF/nif.owl). The nif.owl file uses approximately 30 external files. The OntoFox method and the OWLAPI modularization method were separately performed and compared. For the OWLAPI modularization, the OWLAPI SyntacticLocalityModuleExtractor with STAR module type was used.

## Results

### MIREOT implementation

The MIREOT guideline suggests the following minimal set: (1) source term URI, (2) target direct superclass URI, and (3) source ontology URI [14]. These are the first parameters taken as input by OntoFox:

#### 1) Source ontology URI

Box 1 of the OntoFox web input system includes a list of the 15 ontologies a user can select as source ontology (Figure 2 and [Additional file 1). Alternatively, a user can request an unlisted source ontology in Box 2, in which case the URL of a SPARQL endpoint where this new source ontology can be accessed must be provided. For each external ontology term, OntoFox adds an *importedFrom *annotation property http://purl.obolibrary.org/obo/IAO_0000412, which indicates the URI of the source ontology.

#### (2) Low level source term URI

This parameter is equivalent to the source term URI in the MIREOT guideline. Box 3 allows users to input one or multiple source term URIs, entering one URI per line. For example: http://purl.org/obo/owl/NCBITaxon#NCBITaxon_9606 #Homo sapiens

#### (3) Target direct superclass URI

This is the URI of the direct superclass of the top-level source term chosen above (*i.e*., where to position the newly imported term(s) in the target ontology). This parameter is entered alongside the top-level source term URI in Box 4 using the directive "subClassOf" (see more detail below).

These three data items together unambiguously define a single term from the source ontology and where to position (*i.e*., what class is it a subclass of) it in the target ontology.

### Annotation properties management

OntoFox provides several settings/directives allowing users to select which annotation properties to retrieve, and more importantly, under which format those should be returned.

#### (1) Source term annotation URIs

By default (*i.e*., if no annotation URI is specified), OntoFox will not fetch any of the annotation properties of the selected term. A user can choose to retrieve specific annotation properties by specifying their URIs, or use the OntoFox command 'includeAllAxioms' to fetch all annotations properties associated with source ontology terms. This parameter is entered in Box 6 in the web input format (Figure 2).

#### (2) "copyTo"

**Figure 3:**
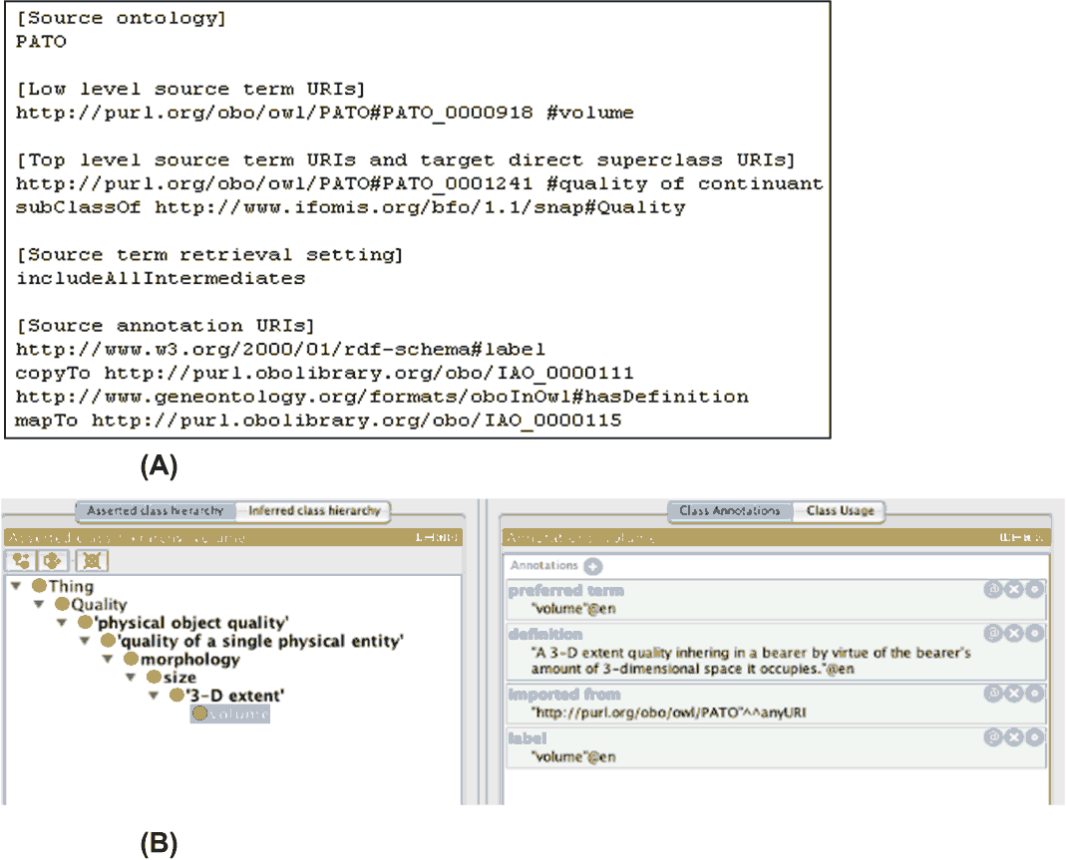
**OntoFox retrieval of PATO term 'volume' and its annotations**. (A) OntoFox input data; (B) Protégé display of OntoFox output data [Additional file 2]. All terms from 'volume' up to 'quality of continuant' in PATO have been imported and positioned under the BFO term Quality. The desired annotation properties (IAO_0000111 'preferred term' and IAO_0000115 'definition') have been specified using OntoFox directives 'copyTo' and 'mapTo'.

This directive is used to map an ontology term annotation to a new annotation property created in the target ontology, resulting in a duplication of the annotation property value in the output file. It is used at the beginning of a line, followed by an annotation URI used in target ontology. For example, the "*copyTo*" command is used in Figure 3A

http://www.w3.org/2000/01/rdf-schema#label

copyTo http://purl.obolibrary.org/obo/IAO_0000111#preferred term

This duplicates the value of the rdfs:label property into the "preferred_term" annotation (IAO_0000111 from IAO [28]), and both annotations are included in the output file. This directive can be used in the web form (Box 6) or in the OntoFox input text file.

#### (3) "mapTo"

This directive allows mapping of an ontology term annotation: it will replace an existing annotation property in the target ontology with the value of another annotation property from the source ontology. It is used at the beginning of a line, followed by an annotation URI from the target ontology. For example, Figure 3 (together with [Additional file 2]) contains an example of using the "mapTo" directive:

http://www.geneontology.org/formats/oboInOwl#hasDefinition

mapTo http://purl.obolibrary.org/obo/IAO_0000115#definition annotation property

As ontologies don't always use a common set of annotation properties, this feature provides an easy way to integrate information from a source ontology into a target ontology while retaining a consistent, metadata style. For example, the OBO2OWL script http://www.berkeleybop.org/obo-conv.cgi, used to automatically generate OWL version of OBO ontologies within the OBO Foundry, uses the property "hasDefinition" to relate a term to an instance whose rdfs:label is that term's definition. However VO uses the IAO metadata scheme http://code.google.com/p/information-artifact-ontology/wiki/OntologyMetadata, and directly relates the term to its definition via the http://purl.obolibrary.org/obo/IAO_0000115, *definition *annotation property. The *mapTo *directive instructs OntoFox to map the definition used in the source to the value of the VO annotation property for definition. This mapping directive is used in Box 6 of the web form input method or in OntoFox input text format.

### Managing incorporation of related terms

OntoFox provides a number of mechanisms for selecting related terms for import, all based on structural approaches and that have been used within VO development. Methods are provided for selective retrieval of parent terms, transitive retrieval of restrictions inspired by structural-based modularization techniques, and the extraction of a subtree rooted at a given term. In this section we detail these mechanisms.

The setting "*Top level source term URI*", is designed to work in conjunction with another term specification when retrieving parent terms between lower and upper level source terms. A typical use is when importing some or all of the superclasses of a species term to allow for queries at different taxonomic ranks *(e.g*., kingdom, phylum, and species). For example, in between '*homo sapiens*' and Eukaryota (our chosen top-level term) in the NCBI Taxonomy, there are 27 intermediate terms (cf Figure 2). It would be very tedious to find, copy and then paste all those 29 terms into the new ontology. By specifying '*homo sapiens*' as the low level source term, Eukaryota as the upper level source term, and the setting "*includeAllIntermediates*", the 27 intermediate terms are automatically retrieved by OntoFox (Figure 2 and [Additional file 1]).

**Figure 4:**
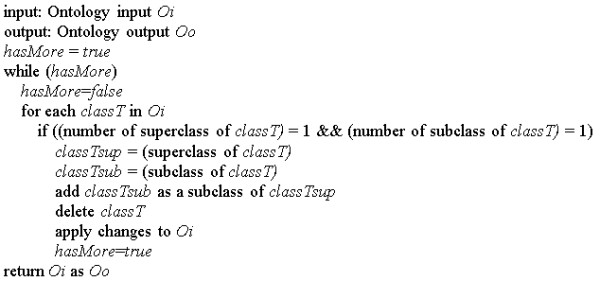
**OntoFox algorithm for extracting computed intermediate classes**. It removes any intermediate classes that have only one parent class and only child class. Only intermediate terms with at least two child classes are kept.

In addition to this retrieval of all parent terms, OntoFox uses an algorithm to compute and retrieve intermediate source terms that are the closest ancestors of more than one low-level source terms, and to remove intermediate terms that have only one parent term and one child term (Figure 4), leaving only terms that present alternatives for query. This setting, "*includeComputedIntermediates*", provides an option to reduce the number of extracted ontology terms by getting less intermediate ontology terms than that with the setting "*includeAllIntermediates*" (Figure 2), while still fulfilling many users' requirement.

**Figure 5:**
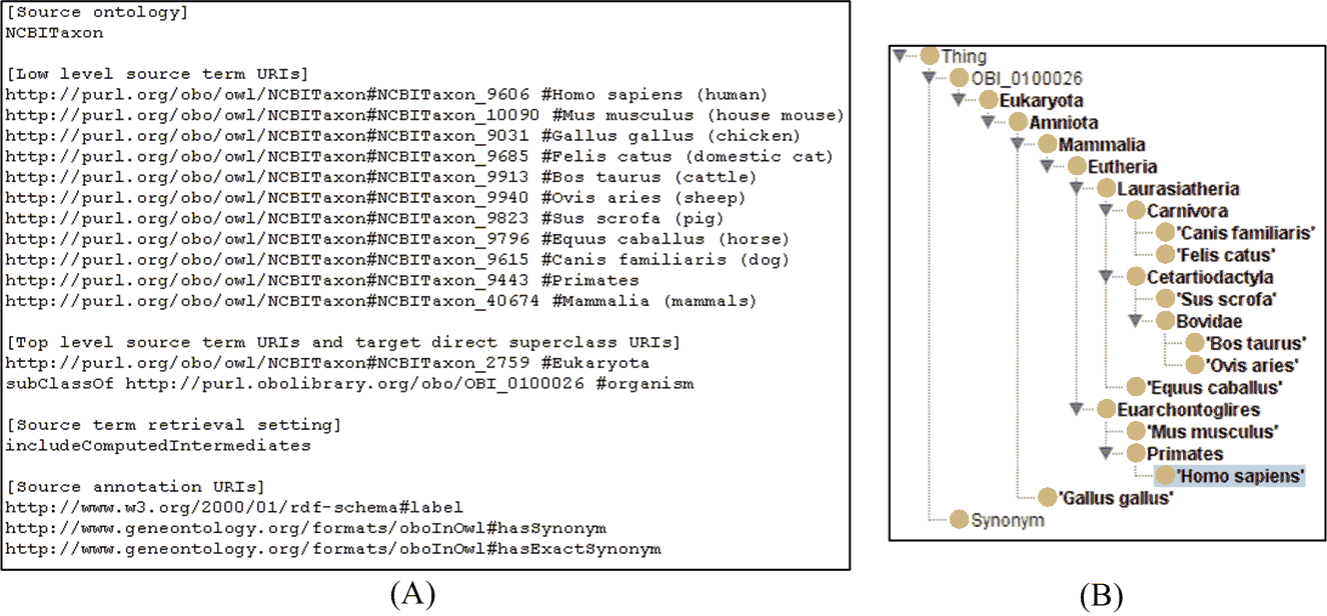
**OntoFox demonstration of the *includeComputedIntermediates *setting**. Terms that are common ancestors (e.g., Bovidae) to at least 2 external terms are kept in the resulting hierarchy, in addition to the terms (e.g., Primates) explicitly requested.

Figure 5 demonstrates the usage of this setting. 11 commonly used animal species are included as the low-level source terms. If we use the setting "*includeAllIntermediates*", 70 intermediate terms will be included. However, only six intermediate terms are included after the "*includeComputedIntermediates*" setting is applied (Figure 5). Each of these six intermediate terms (*e.g*., Euarchontoglires) is the immediate parent class for at least two child terms (*e.g*., Primates and Homo sapiens). Primates and mammals are not leaf nodes in the taxonomy hierarchy when the sole parent term is Homo sapiens. Since we wanted these terms as well in the final result of the OntoFox output file, we have intentionally included them as low-level source terms.

**Figure 6:**
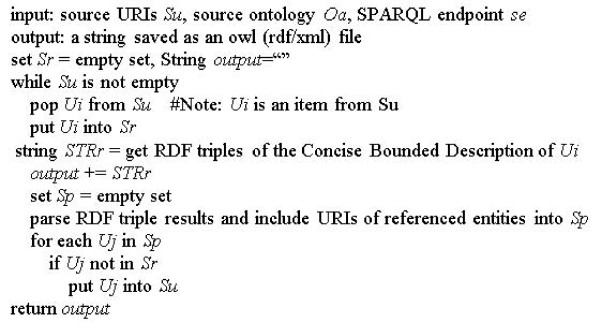
**OntoFox SPARQL-based algorithm for retrieval of related terms**. Its goal is to extract related terms and annotations associated with a set of signature terms (stored in *Su*) from an external ontology. This method was performed in OntoFox when the setting "*includeAllAxioms*" is selected.

A third choice for including selected terms is inspired by structural modularization techniques. Given a set of signature terms, OntoFox retrieves restrictions that are parent classes of a term. This choice is implemented using OntoFox's SPARQL-based related term retrieval algorithm (Figure 6). Where a restriction mentions another class, restrictions on that class are queried, and so on, until a fixed point is reached. The method gives useful results with the ontologies at the typical level of complexity we encounter. It also has the benefit of being straightforwardly implemented in SPARQL. SPARQL is able to easily retrieve the Concise Bounded Description (CBD) of a resource by means of an RDF graph, which is a general and broadly optimal unit of specific knowledge about that resource to be utilized by, and/or interchanged between, semantic web agents [29]. Current modularization algorithms use in-memory representations that require excessive memory for ontologies such as NCBI Taxonomy. In contrast, the SPARQL-based approach is highly scalable. Within the OntoFox user interface, users select this choice by choosing "*includeAllAxioms"*.

To test OntoFox's SPARQL method to retrieve related terms, three sets of signature terms (individual term, small subset of terms, larger ontology file) were given as input to the OntoFox method and OWLAPI modularization method. In all three cases, both methods generated identical results. One comparative test was performed using the set of terms in the Neurodegenerative Disease Phenotype Ontology (NDPO; http://ccdb.ucsd.edu/NDPO/1.0/NDPO.owl) that imports the Neuroscience Information Framework (NIF) Lexicon ontology (nif.owl; http://ontology.neuinfo.org/NIF/nif.owl). The imports closure of this OWL file contains some 50,000 classes in 87 MB of OWL files. Applying the OWLAPI to the classes and object properties in NDPO.owl yielded a module with 1351 classes and 7 object properties - roughly 2.5 M OWL file including annotations. The OntoFox generated the same results as measured by the ontology metrics provided by Protégé 4.1. These results support our claim that the OntoFox approach is an effective method for extracting related ontology terms.

Finally OntoFox can extract the whole branch ontology terms below a specific ontology term (Box 5 in Figure 1).

Choices such as which terms in a parent hierarchy should be included are preliminary to module extraction techniques, which take as input a set of terms (signature) that the ontology developer has identified as being of interest. OntoFox supports experimentation by offering more than one choice for making such a term selection.

### OntoFox data input and result output

Besides the web form-based data input, data can be uploaded as a text file to the OntoFox web server. This input file contains the same information as the web form input method, but makes it easier to submit batch jobs. The file upload method also makes it possible to keep track of submissions and easily update the input. An OntoFox sample input file (available at http://ontofox.hegroup.org/format.txt) has been developed for users to quickly understand and use the required format. Also, the OntoFox input file can also be automatically generated using the button "Generate OntoFox Input File" from data in the web forms (Figure 2).

Finally, jobs can be programmatically submitted to the OntoFox server via a script at http://ontofox.hegroup.org/service.php. As an example, the following command line can be used to provide an input file (input.txt) and retrieve the corresponding output file (output.owl): "curl -s -F file = @/tmp/input.txt -o/tmp/output.owl http://ontofox.hegroup.org/service.php.

An OntoFox query can result in either a processing error, in which case an explicit message is provided to the user, or in the production of an OWL file serialized in the RDF/XML format. This OWL file constitutes an ontology on its own and can be visualized using the Protégé ontology editor [11] and directly imported into the target ontology using the OWL import directive.

The OntoFox process can be executed at different times to import updated information of external ontology terms. By keeping and updating the original OntoFox input text file, users can subsequently query the OntoFox server on a regular basis and get up to date information with little effort.

### OntoFox application in Vaccine Ontology (VO) development

Using OntoFox, VO currently imports approximately 1000 terms from 12 external ontologies such as GO [1], NCBI Taxonomy, OBI, PATO (Phenotypic Quality Ontology) [30], and MP (Mammalian Phenotype Ontology) [31] (Table 2).

**Table 2:** OntoFoxed ontologies in VO

#	Ontology Name	# of signature terms	# of imported terms	Intermediates	Annotations
1	CARO	2	2	No	rdfs:label *copyTo *iao:preferredTerm oboInOwl:hasDefinition *copyTo *iao:definition oboInOwl:hasSynonym *mapTo *iao:alternativeTerm
	
2	CHEBI	13	13	No	
	
3	DOID	10	57	All	
	
4	FMA	2	2	No	
	
5	GO	2	2	No	
	
6	IDO	1	2	No	
	
7	NCBITaxon	143	198	Computed	

8	OBI	41	48	All	rdfs:label iao:definition

9	PATO	15	17	Computed	rdfs:label *copyTo *iao:preferredTerm oboInOwl:hasDefinition *copyTo *iao:definition oboInOwl:hasSynonym *mapTo* iao:alternativeTerm
	
10	PRO	2	2	Computed	
	
11	ro_proposed	7	9	All	
	
12	SO	1	1	No	

When using OntoFox to develop VO, we found desirable to apply different settings depending on the source ontology considered, and therefore generated one OWL file to be imported per external resource. Once imported into VO, external terms can be used exactly in the same way as other vaccine-specific VO terms.

Different OntoFox settings have been applied for generating these 12 ontology subsets for VO imports (Table 2). In terms of superclass extraction, six were generated with the OntoFox setting "*includeNoIntermediates*", which is particularly useful when the intermediate superclasses do not generate much more information needed for the target ontology. The setting "*includeComputedIntermediates*" was used for extracting ontology terms from three external resources, including NCBITaxon, PATO, and PRO [32]. In the case of the NCBI taxonomy it reduces the number of imported classes without losing the information of the most recent ancestor superclasses (Figure 5). Finally, the setting "*includeAllIntermediates*" has been used for extraction from OBI, ro_proposed http://purl.org/obo/owl/ro_proposed, and the Sequence Ontology (SO; http://www.sequenceontology.org/) (Table 2). These three external ontologies are closely related to VO, and we would like to use all original hierarchies for those terms imported to VO.

Similarly, different annotation property settings have been applied (Table 2). Typically, VO follows the IAO's ontology metadata scheme and uses the properties "rdfs:label" or "iao:definition". To make the annotation styles consistent among all ontology terms in VO, the OntoFox directives "copyTo" and "mapTo" were used (Table 2).

## Discussion

While an implementation of the MIREOT strategy has been performed in the context of OBI, it relies on command line scripts, making it use impractical for the average ontology curator and limiting its adoption by interested users. Comparatively, OntoFox provides a convenient web-based approach to use MIREOT that does not require programmatic skills and allows users to specify their requirements via simple text formats.

In addition, the OntoFox server provides additional options for users to add and rewrite annotations, to include superclasses or subclasses, or select terms via related restrictions (transitively). This last option performs comparable to existing structural modularization methods.

OntoFox uses a RDF triple store and SPARQL for information storage and retrieval, resulting in a system that scales better than in-memory modularization techniques. For the Neurodegenerative Disease Phenotype Ontology, OntoFox extracted the same module that a more sophisticated modularization technique did. While we don't argue that these more sophisticated techniques are not desirable, we do note that the authors acknowledge there are issues with their use. While OntoFox uses simpler methods to retrieve terms and axioms related to MIREOT specified terms, it provides a simpler and more understandable approach to reuse. This is particularly useful in conjunction with the fact that OntoFox provides an easy approach to incorporate frequent updates from source ontologies that are under active development.

The provision of a simple mechanism for importing selected terms from external ontologies does not shield the user from general issues associated with using external terms. When using terms from other ontologies, care must be taken to avoid a situation in which the meaning of an ontology term in the source ontology is different from the meaning of the term used in the target ontology. To avoid this problem, users are advised to exercise due diligence when selecting terms to import. OntoFox helps prevent this confusion, by first offering a limited set of 15 selected ontologies with good documentation and second by importing annotation properties, providing immediate access to the textual definitions. Where an ontology developer has questions as to the meaning of a term we recommend that they contact the developers of the source ontology and ask for clarification and enhanced documentation. Our 15 initially selected ontologies generally have trackers and mailing lists where questions can be posted.

Another issue is the evolution problem associated with using ontologies that are under active development, as is the case with most current biomedical ontologies. Although at a certain time point a certain term is used in the source and target ontologies equivalently, over time the usage of the term (and the associated classes) in both ontologies may change. It is considered good practice to not use terms from external ontologies in ways not consistent with their definition, and for ontologies to deprecate old and define new terms rather than changing the meaning of terms. While OntoFox provides a way for users to automatically update the annotations of imported terms, it cannot monitor changes in meaning. Therefore it is up to developers to choose ontologies that have practice that will let them monitor for such changes and make adjustments as appropriate. OntoFox's 15 initial ontologies were chosen because they tend to have predictable practices related to ontology evolution.

## Conclusions

The web-based OntoFox system supports ontology reuse by following and extending the existing MIREOT principle and developing a new ontology term retrieval method. It dynamically extracts external ontology terms and their chosen annotations from SPARQL endpoint(s), while not requiring any prior knowledge of the SPARQL query language and/or computational programming skills. Finally, OntoFox provides flexible controls over management and customization of the import.

Further development is planned. For example, we will include more ontologies in our list of source ontology repositories. These ontologies may come from the OBO foundry or other reliable sources. We are also considering developing an OntoFox plugin for ontology editors (e.g., Protégé). Editors of OBO format ontologies desire a similar facility, and while OntoFox currently supports resources in OWL, we plan to support the OBO format directly by integrating an automatic conversion for OBO files. As usability testing of our web interface has thus far been informal, we will perform more careful usability studies and have designed a survey to solicit feedback from the community.

With more ontologies being developed, OntoFox offers a timely web-based package supporting efficient ontology reuse.

## Availability and requirements

The OntoFox web system is freely available at: http://ontofox.hegroup.org/. Its usage requires Internet access. The source code is also freely available under the Apache License 2.0 [Additional file 3].

## List of Abbreviations

API: Application Programming Interface; BFO: Basic Formal Ontology; CHEBI: Chemical Entities of Biological Interest; GO: Gene Ontology; FMA: Foundational Model of Anatomy; InfluenzO: The Influenza Ontology; PATO: Phenotypic Quality Ontology; MIREOT: Minimum information to reference external ontology terms; MP: Mammalian Phenotype Ontology; NCBI: The National Center for Biotechnology Information; NCBO: The National Center for Biomedical Ontology; NEMO: Neural ElectroMagnetic Ontologies; NIF: The Neuroscience Informatics Framework; OBI: Ontology for Biomedical Investigations; OBO: The Open Biomedical Ontologies; OWL: Web Ontology Language; PATO: Phenotypic Quality Ontology; PHP: Hypertext Preprocessor; PRO: Protein Ontology; RO: OBO Relation Ontology; ro_proposed: RO proposed version; SPARQL: SPARQL Protocol and RDF Query Language; SO: Sequence Ontology; RDF: Resource Description Framework; URI: Uniform Resource Identifier; VO: Vaccine Ontology.

## Competing interests

The authors declare that they have no competing interests.

## Authors' contributions

ZX: Webmaster, software programmer, database administrator, use case testing, and manuscript editing. MC: OntoFox testing, discussion, valuable feedback and suggestions, and manuscript editing. RRB: Discussion, valuable feedback and suggestions, and manuscript editing. AR: OntoFox testing, discussion, valuable feedback and suggestions, and manuscript editing. YH: Project design and management, programming, use case testing, and drafting of manuscript.

## Supplementary Material

Additional file 1 **Output file for Figure **2. This is the output file in OWL (RDF/XML) format based on the OntoFox input shown in Figure 2B. Click here for file

Additional file 2 **Output file for Figure **3. This is the output file used in OWL (RDF/XML) format based on the OntoFox input shown in Figure 3A.
Click here for file

Additional file 3 **The source code of the OntoFox software**. This zip file includes PHP source code of the OntoFox website and the Java source code of for reformatting/trimming owl (RDF/XML) output file. Click here for file
