# Supplementary material for: OntoFox: web-based support for ontology reuse
Source: BMC Res Notes. 2010 Jun 22;3:175. doi: 10.1186/1756-0500-3-175 (PMC2911465; doi:10.1186/1756-0500-3-175)
Supplement: Additional file 3 — The source code of the OntoFox software. This zip file includes PHP source code of the OntoFox website and the Java source code of for reformatting/trimming owl (RDF/XML) output file. [file 1756-0500-3-175-S3.ZIP › website/acknowledge.php]

OntoFox


HomeIntroductionTutorialFAQsReferencesLinksContactAcknowledge

### Acknowledgements

This research is supported by a Rackham Pilot Research grant to Dr. Yongqun "Oliver" He at the University of Michigan. We appreciate suggestions provided by Melanie Courtot and Alan Ruttenberg from the OBI Consortium.

|  |  |
| --- | --- |
| He Group  University of Michigan Medical School  Ann Arbor, MI 48109 |  |
