# Supplementary material for: OntoFox: web-based support for ontology reuse
Source: BMC Res Notes. 2010 Jun 22;3:175. doi: 10.1186/1756-0500-3-175 (PMC2911465; doi:10.1186/1756-0500-3-175)
Supplement: Additional file 3 — The source code of the OntoFox software. This zip file includes PHP source code of the OntoFox website and the Java source code of for reformatting/trimming owl (RDF/XML) output file. [file 1756-0500-3-175-S3.ZIP › website/contactus.php]

OntoFox


HomeIntroductionTutorialFAQsReferencesLinksContactAcknowledge

### Contact Us

The OntoFox system is developed by He Group at the University of Michigan, Ann Arbor, MI. Please contact us:

> Zuoshuang Xiang: Software Developer, Database Administrator, and Webmaster. University of Michigan Medical School   
> Email:    
> Phone: (734) 615-2455

> Dr. Yongqun "Oliver" He, Assistant Professor, University of Michigan Medical School   
> Email:    
> Phone: (734) 615-8231

You can also send your suggestions and comments via the online Feedback Form.

If you have questions about MIREOT, you can contact OBI MIREOT project.

Your suggestions and comments are welcome. Thank you.

|  |  |
| --- | --- |
| He Group  University of Michigan Medical School  Ann Arbor, MI 48109 |  |
