# Supplementary material for: OntoFox: web-based support for ontology reuse
Source: BMC Res Notes. 2010 Jun 22;3:175. doi: 10.1186/1756-0500-3-175 (PMC2911465; doi:10.1186/1756-0500-3-175)
Supplement: Additional file 3 — The source code of the OntoFox software. This zip file includes PHP source code of the OntoFox website and the Java source code of for reformatting/trimming owl (RDF/XML) output file. [file 1756-0500-3-175-S3.ZIP › website/faqs.php]

OntoFox


HomeIntroductionTutorialFAQsReferencesLinksContactAcknowledge

### Frequently Asked Questions

**1. What is OntoFox?**

OntoFox is a web server that automatically extract annotations and intermediate layers of bio-ontologies.

**2.** **Who are primary users of OntoFox?**

Bio-ontology developers, and bioinformaticians who are using bio-ontologies for different applications.

**3.** **What is URI?**

A Uniform Resource Identifier (URI) is defined in [RFC3986] as a sequence of characters chosen from a limited subset of the repertoire of US-ASCII [ASCII] characters. URIs refer to resources.

**4.** **Where are the source ontologies used in OntoFox stored?**

OntoFox uses source ontologies stored in RDF format in Neurocommons and a RDF server in He Group in University of Michigan Medical School. The contents of source ontologies are stored in RDF triples and available for SPARQL query. Theoretically, OntoFox is able to fetch any ontology in a SPARQL queriable RDF server through internet.

**5.** **How can we use OntoFox to get a single class from source ontology without including a hierarchy of superclasses?**

To get a single class using OntoFox, you can specify the top level source term URI the same as the low level source term URI. Or , you don't need to specify any top level source term URI. By default, OntoFox fetches a single class unless a top level superclass is specified.

**6.** **Can we use OntoFox for ontologies developed using OBO format?**

OntoFox is developed based on OWL format. Currently there are many converters that can convert OBO format to OWL format or vice versa. With the help of some converter, it is possible to use OntoFox for development of a new ontology based on OBO format.

**7.** **How can I use OntoFox to add new terms and update annotations?**

It is recommended that you run OntoFox again when you like to update your annotations. You may consider to keep one master OntoFox input file. Whenever you like to add new terms from external ontologies, you can update the OntoFox input file by adding new term URIs and other related information, and then rerun OntoFox using our file upload option.

**8.** **Who has used OntoFox for ontology development? Any successful stories?**

OntoFox has been routinely used for Vaccine Ontology (VO) development. We have also tested OntoFox successfully for importing external ontology terms for development of the Ontology for Biomedical Investigations (OBI).

**9.** **Why are Basic Formal Ontology (BFO) and Relation Ontology (RO) imported as a whole, rather than being included in OntoFox as source ontologies for mireoting?**

BFO is the upper level ontology which have been adopted by many domain ontologies developed for scientific research, including those in the OBO Foundry. Similarly, RO is an ontology of core relations used by OBO Foundry ontologies and many other ontologies. Both BFO and RO have relatively small sizes but are essential for ontology development. If you use BFO and RO, you would like to import them as a whole. Therefore, we don't include BFO and RO as source ontologies for OntoFox mireoting.

**10.** **How are the input and output files provided by the users stored on OntoFox servers?**

The input and output files are not stored permanently on the OntoFox servers. They will be stored for up to 24 hours with a unique file name which consists of 8 random characters. These files will be automatically destroyed at 3:00 am EST (New York time) by an internal script. The temporary storage is for users to come back to the input and result files, and it also provides a way for our OntoFox developers to debug any possible errors. For those users who do have concerns on privacy and security, the users can select to destroy the input and output files immediately in the end of the OntoFox execution.

**11. Is it**  **possible to enter a favorite source ontology and SPARQL end point and then select a different ontology via the drop-down menu?**

We do not allow the option of using a favorite source ontology and SPARQL endpoint and at the same time using a different ontology via the drop-down menu. OntoFox prevents a user from providing two ontology sources. Specifically, when a favorite source ontology and SPARQL endpoint are provided, the drop-down menu does not show any specific ontology. If a different ontology is selected from the drop-down menu, any text in the favorite source ontology input box will automatically be cleared.

**12: Is it possible to access OntoFox programmatically?**

Yes. To access OntoFox without using the OntoFox web page, try this: curl -s -F file=@/tmp/input.txt -o /tmp/output.owl http://ontofox.hegroup.org/service.php  
See more information in Tutorial.

|  |  |
| --- | --- |
| He Group  University of Michigan Medical School  Ann Arbor, MI 48109 |  |
