# Supplementary material for: OntoFox: web-based support for ontology reuse
Source: BMC Res Notes. 2010 Jun 22;3:175. doi: 10.1186/1756-0500-3-175 (PMC2911465; doi:10.1186/1756-0500-3-175)
Supplement: Additional file 3 — The source code of the OntoFox software. This zip file includes PHP source code of the OntoFox website and the Java source code of for reformatting/trimming owl (RDF/XML) output file. [file 1756-0500-3-175-S3.ZIP › website/getExternal.php]

OntoFox


HomeIntroductionTutorialFAQsReferencesLinksContactAcknowledge

Retrieving Results

/\*\*
\* Author: Zuoshuang Xiang
\* The University Of Michigan
\* He Group
\* Date: 2010-03-04
\*
\* This is the main program for processing user inputs, form queries,
\* process query resuslt and output final results.
\*/
set\_time\_limit(60\*60);
include('inc/functions.php');
$vali=new Validation($\_REQUEST);
$outputURI = '';
$outputFile = 'import.owl';
$finalFile = createRandomPassword();
$str\_inputs = array();
//If user upload a file, parse the file and seperate into individual ontologies.
if (isset($\_FILES['file']) && is\_uploaded\_file($\_FILES['file']['tmp\_name'])){
$data = trim(file\_get\_contents($\_FILES['file']['tmp\_name']));
file\_put\_contents("$userfiles/$finalFile.txt", $data);
if (preg\_match('/\_input\.txt$/', $\_FILES['file']['name'])) {
$outputFile = preg\_replace('/\_input\.txt/', '.owl', $\_FILES['file']['name']);
$outputURI = "http://purl.obolibrary.org/obo/vo/external/$outputFile";
}
$lines = preg\_split('/[\r\n]+/', $data);
$str\_input='';
foreach($lines as $line) {
if (strpos($line, ' #')!==false) {
$line = trim(substr($line, 0, strpos($line, ' #')));
}
if (strpos($line, '#')===0) {
//ignore comments
}
elseif (strpos($line, '[Source ontology]')===0) {
if (strpos($str\_input, '[Source ontology]')===0) {
$str\_inputs[] = $str\_input;
}
$str\_input = '[Source ontology]';
}
else {
$str\_input .= "\n" . $line;
}
}
if (strpos($str\_input, '[Source ontology]')===0) {
$str\_inputs[] = $str\_input;
}
}
else {
$ontology= $vali-getInput('ontology', 'Ontology', 0, 128);
$ontology2 = $vali->getInput('ontology2', 'Your own ontology', 0, 1024);
$ontology = trim($ontology . "\n" . $ontology2);
$term\_iris = $vali->getInput('term\_iris', 'Lower level term IRIs to be imported', 0, 81920);
$top\_term\_iris = $vali->getInput('top\_term\_iris', 'Top level term IRIs to be imported', 0, 8192);
$top\_term\_iris2 = $vali->getInput('top\_term\_iris2', 'Top level term IRIs to be imported', 0, 8192);
$retrieval\_setting = $vali->getInput('retrieval\_setting', 'Source term retrieval setting', 0, 128);
$annotation\_iris = $vali->getInput('annotation\_iris', 'annotation IRIs to be included', 0, 8192);
$str\_inputs[] = "[Source ontology]
$ontology
[Low level source term URIs]
$term\_iris
[Top level source term URIs and target direct superclass URIs]
$top\_term\_iris
[Source term retrieval setting]
$retrieval\_setting
[Branch extractions from source term URIs and target direct superclass URIs]
$top\_term\_iris2
[Source annotation URIs]
$annotation\_iris";
file\_put\_contents("$userfiles/$finalFile.txt", $str\_inputs[0]);
}
$fileNames=array();
$str\_inputs2=array();
foreach ($str\_inputs as $str\_input) {
$lines = preg\_split('/[\r\n]+/', $str\_input);
$current\_tag = '';
foreach ($lines as $line) {
$line = trim($line);
if (strpos($line, '[')===0) {
foreach($section\_tags as $section\_tag\_var => $section\_tag\_txt){
if (strpos($line, $section\_tag\_txt)===0) {
$current\_tag = $section\_tag\_var;
eval("$current\_tag = '';");
}
}
}
elseif (strpos($line, '#')===0) {
//ignore comments
}
else {
if (strpos($line, ' #')!==false) {
$line = trim(substr($line, 0, strpos($line, ' #')));
}
if ($current\_tag!='' && $line!='') {
eval("$current\_tag .= \"$line\\n\";");
}
}
}
if ($term\_iris!=='') {
$str\_inputs2[] = "[Source ontology]
$ontology
[Low level source term URIs]
$term\_iris
[Top level source term URIs and target direct superclass URIs]
$top\_term\_iris
[Source term retrieval setting]
$retrieval\_setting
[Source annotation URIs]
$annotation\_iris";
}
if ($top\_term\_iris2!=='') {
$str\_inputs2[] = "[Source ontology]
$ontology
[Top level source term URIs and target direct superclass URIs]
$top\_term\_iris2
[Source annotation URIs]
$annotation\_iris";
}
}
foreach ($str\_inputs2 as $str\_input) {
$lines = preg\_split('/[\r\n]+/', $str\_input);
$current\_tag = '';
foreach ($lines as $line) {
$line = trim($line);
if (strpos($line, '[')===0) {
foreach($section\_tags as $section\_tag\_var => $section\_tag\_txt){
if (strpos($line, $section\_tag\_txt)===0) {
$current\_tag = $section\_tag\_var;
eval("$current\_tag = '';");
}
}
}
elseif (strpos($line, '#')===0) {
//ignore comments
}
else {
if (strpos($line, ' #')!==false) {
$line = trim(substr($line, 0, strpos($line, ' #')));
}
if ($current\_tag!='' && $line!='') {
eval("$current\_tag .= \"$line\\n\";");
}
}
}
if (!isset($term\_iris)) $term\_iris='';
if (!isset($top\_term\_iris)) $top\_term\_iris='';
if (strlen($term\_iris)<10 && strlen($top\_term\_iris)<10) {
$vali->concatError("At least one lower level term IRI is required for individual term extraction or at least one top level term IRI is required for branch extraction.");
}
if (!isset($ontology) || strlen($ontology)<2) {
$vali->concatError("Ontology is required.");
}
//Get source ontology
if ($vali->getErrorMsg()=='') {
$extractBranch=false;
if ($term\_iris=='') {
$extractBranch=true;
}
//parse ontology
$lines = preg\_split('/[\r\n]+/', trim($ontology));
$ontology\_uri = '';
$ontology\_original\_uri='';
$server\_import = '';
foreach ($lines as $line) {
$line = trim($line);
if (strpos($line, '#')===0) {
//ignore comments
}
else {
if (strpos($line, ' #')!==false) {
$line = trim(substr($line, 0, strpos($line, ' #')));
}
if (strpos($line, 'fromEndpoint ')===false) {
$ontology = $line;
if (isset($array\_ns[$line])) {
$ontology\_uri = $array\_ns[$line];
$ontology\_original\_uri = $array\_original\_ns[$line];
$server\_import = $array\_server[$line];
}
else {
$ontology\_uri = $line;
$ontology\_original\_uri= $line;
}
}
else {
$line = trim(str\_replace('fromEndpoint ', '', $line));
$server\_import = $line;
}
}
}
$retrieval\_setting = isset($retrieval\_setting) ? trim($retrieval\_setting) : '';
if ($retrieval\_setting=='') {
$retrieval\_setting='includeNoIntermediates';
}
if ($extractBranch) {
//for branch extraction
$retrieval\_setting='includeAllIntermediates';
}
$outputNSs = array();
$outputNSs['http://www.w3.org/1999/02/22-rdf-syntax-ns#'] = 'rdf';
$outputNSs['http://www.w3.org/2002/07/owl#'] = 'owl';
$outputNSs['http://purl.obolibrary.org/obo/'] = 'obo';
$outputNSs['http://www.w3.org/2000/01/rdf-schema#'] = 'rdfs';
$strOutput='>
imported from
';
$ns\_rdf = 'http://www.w3.org/1999/02/22-rdf-syntax-ns#';
$ns\_rdfs = 'http://www.w3.org/2000/01/rdf-schema#';
$ns\_owl = 'http://www.w3.org/2002/07/owl#';
$included\_iris = array();
$parent\_included\_iris = array();
$terms\_to\_keep = array();
$top\_term\_iris=trim($top\_term\_iris);
//By default, don't retrive structure.
if ($top\_term\_iris=='') {
$top\_term\_iris = $term\_iris;
}
//Special case to help lazy people.
if ((strpos($top\_term\_iris, 'subClassOf ')===0 || strpos($top\_term\_iris, 'subPropertyOf ')===0 || strpos($top\_term\_iris, 'type ')===0) && !preg\_match('/[\r\n]+/', $top\_term\_iris)) {
$top\_term\_iris = preg\_replace('/[\r\n]+/', "\n" . $top\_term\_iris . "\n", $term\_iris) . "\n" . $top\_term\_iris;
}
//add top level source terms
$lines = preg\_split('/[\r\n]+/', trim($top\_term\_iris));
$current\_iri ='';
foreach ($lines as $line) {
$line = trim($line);
if (strpos($line, '#')===0) {
//ignore comments
}
else {
if (strpos($line, ' #')!==false) {
$line = trim(substr($line, 0, strpos($line, ' #')));
}
if (strpos($line, 'subClassOf ')===0) {
$line = trim(str\_replace('subClassOf ', '', $line));
if ($current\_iri!='') {
$terms\_to\_keep[$line] = 1;
$strOutput.="


";
}
}
elseif (strpos($line, 'subPropertyOf ')===0) {
$line = trim(str\_replace('subPropertyOf ', '', $line));
if ($current\_iri!='') {
$terms\_to\_keep[$line] = 1;
$strOutput.="


";
}
}
elseif (strpos($line, 'type ')===0) {
$line = trim(str\_replace('type ', '', $line));
if ($current\_iri!='') {
$terms\_to\_keep[$line] = 1;
$strOutput.="


";
}
}
else {
$parent\_included\_iris[$line] = 'NA';
$current\_iri=$line;
$terms\_to\_keep[$line] = 1;
}
}
}
//Get annotation URLs user wish to include
$annotation\_iris\_to\_include = array();
$annotation\_iris\_to\_include['http://www.w3.org/1999/02/22-rdf-syntax-ns#type'] = array('action'=>'', 'iri'=>'');
if (!$extractBranch) {
$annotation\_iris\_to\_include['http://www.w3.org/2000/01/rdf-schema#subClassOf'] = array('action'=>'', 'iri'=>'');
$annotation\_iris\_to\_include['http://www.w3.org/2000/01/rdf-schema#subPropertyOf'] = array('action'=>'', 'iri'=>'');
$annotation\_iris\_to\_include['http://www.w3.org/2002/07/owl#equivalentClass'] = array('action'=>'', 'iri'=>'');
}
if ($annotation\_iris!='') {
$lines = preg\_split('/[\r\n]+/', trim($annotation\_iris));
$current\_iri ='';
foreach ($lines as $line) {
$line = trim($line);
if (strpos($line, '#')===0) {
//ignore comments
}
else {
if (strpos($line, ' #')!==false) {
$line = trim(substr($line, 0, strpos($line, ' #')));
}
//get all properties
if ($line=='includeAllAxioms') {
$annotation\_iris\_to\_include = array();
break;
}
if(strpos($line, 'copyTo ')!==false) {
$line = trim(str\_replace('copyTo ', '', $line));
$annotation\_iris\_to\_include[$current\_iri] = array('action'=>'copyTo', 'iri'=>$line);
$strOutput.=" \n";
}
elseif(strpos($line, 'mapTo ')!==false) {
$line = trim(str\_replace('mapTo ', '', $line));
$annotation\_iris\_to\_include[$current\_iri] = array('action'=>'mapTo', 'iri'=>$line);
$strOutput.=" \n";
}
else {
$annotation\_iris\_to\_include[$line] = array('action'=>'', 'iri'=>'');
$current\_iri= $line;
}
}
}
}
$imported\_ontologies = array();
include\_once('arc/ARC2.php');
/\* configuration \*/
$config = array(
/\* remote endpoint \*/
'remote\_store\_endpoint' => $server\_import,
);
/\* instantiation \*/
$store = ARC2::getRemoteStore($config);
$num\_queries = 0;
$unprocessed\_iris = array();
$processed\_iris = array();
//print\_r($parent\_included\_iris);
if ($extractBranch) {
foreach ($parent\_included\_iris as $import\_term=>$tmpLabel) {
$terms\_to\_keep[$import\_term] =1;
if (!isset($processed\_iris[$import\_term]) && !isset($included\_iris[$import\_term])) {
$results = array($import\_term => 'NA');
getSubClassAndProperty($ontology\_uri, $results, $import\_term);
foreach ($results as $term\_iri => $term\_label) {
if (!isset($processed\_iris[$term\_iri])) {
$unprocessed\_iris[$term\_iri] = $term\_label;
}
}
}
}
}
else {
//process term URIs to be imported and get super classes/properties.
$lines = preg\_split('/[\r\n]+/', trim($term\_iris));
foreach ($lines as $line) {
if (strpos($line, '#')===0) {
//ignore comments
}
else {
if (strpos($line, ' #')!==false) {
$line = trim(substr($line, 0, strpos($line, ' #')));
}
$import\_term=trim($line);
$terms\_to\_keep[$import\_term] =1;
if (!isset($processed\_iris[$import\_term]) && !isset($included\_iris[$import\_term])) {
$results = array($import\_term => 'NA');
if (!isset($parent\_included\_iris[$import\_term])) {
getSupClassAndProperty($ontology\_uri, $results, $import\_term);
//error\_log("!!!\n", 3, '/tmp/error.log');
foreach ($results as $term\_iri => $term\_label) {
if (!isset($processed\_iris[$term\_iri])) {
$unprocessed\_iris[$term\_iri] = $term\_label;
}
}
}
else {
if (!isset($processed\_iris[$import\_term])) {
$unprocessed\_iris[$import\_term] = 'NA';
}
}
}
}
}
}
//Send query to servers and process response.
while (!empty($unprocessed\_iris)) {
$tmp\_results = array();
$num\_terms\_per\_query = 20;
if (!empty($annotation\_iris\_to\_include)) {
$num\_terms\_per\_query = round($num\_terms\_per\_query/sizeof($annotation\_iris\_to\_include));
if ($num\_terms\_per\_query <2) $num\_terms\_per\_query =2;
}
$array\_iris = array\_chunk($unprocessed\_iris, $num\_terms\_per\_query, true);
foreach ($array\_iris as $iris) {
$querystring = "
CONSTRUCT {
";
$i = 0;
foreach ($iris as $tmp\_iri=>$tmp\_label) {
if (empty($annotation\_iris\_to\_include) || strpos($tmp\_iri, 'nodeID')!==false) {
$i++;
$querystring .= " <$tmp\_iri> ?p{$i} ?o{$i}.
";
}
else {
foreach ($annotation\_iris\_to\_include as $annotation\_iri => $mapping) {
$i++;
if (!preg\_match('/nodeID:/', $tmp\_iri) && $mapping['action']=='copyTo') {
$querystring .= " <$tmp\_iri> <$annotation\_iri> ?o{$i}.
";
$querystring .= " <$tmp\_iri> <{$mapping['iri']}> ?o{$i}.
";
}
elseif (!preg\_match('/nodeID:/', $tmp\_iri) && $mapping['action']=='mapTo') {
$querystring .= " <$tmp\_iri> <{$mapping['iri']}> ?o{$i}.
";
}
else {
$querystring .= " <$tmp\_iri> <$annotation\_iri> ?o{$i}.
";
}
}
}
}
$querystring .= "
}
FROM <$ontology\_uri>
WHERE {
";
$i = 0;
foreach ($iris as $tmp\_iri=>$tmp\_label) {
if (empty($annotation\_iris\_to\_include) || strpos($tmp\_iri, 'nodeID')!==false) {
$i++;
if ($i>1) $querystring .= " UNION";
$querystring .= "
{<$tmp\_iri> ?p{$i} ?o{$i}}
";
}
else {
foreach ($annotation\_iris\_to\_include as $annotation\_iri=>$mapping) {
$i++;
if ($i>1) $querystring .= " UNION";
if (!preg\_match('/nodeID:/', $tmp\_iri) && $mapping['action']=='copyTo') {
$querystring .= "
{<$tmp\_iri> <$annotation\_iri> ?o{$i}}
";
}
elseif (!preg\_match('/nodeID:/', $tmp\_iri) && $mapping['action']=='mapTo') {
$querystring .= "
{<$tmp\_iri> <$annotation\_iri> ?oa{$i} .
?oa{$i}  ?o{$i}}
";
}
else {
$querystring .= "
{<$tmp\_iri> <$annotation\_iri> ?o{$i}}
";
}
}
}
}
$querystring .= "
}";
$querystring = formatQuery($querystring);
//print("");
foreach ($iris as $tmp\_iri => $label) {
$processed\_iris[$tmp\_iri] = $label;
}
$fields = array();
$fields['default-graph-uri'] = '';
$fields['format'] = 'application/rdf+xml';
$fields['debug'] = 'on';
$fields['query'] = $querystring;
$rdf = curl\_post\_contents($server\_import, $fields);
$rdf = preg\_replace('/rdf:nodeID="b/', 'rdf:nodeID="', $rdf);
if (preg\_match\_all('//', $rdf, $matches)){
$lines=$matches[0];
$num\_lines = sizeof($lines);
for($i=$num\_lines-1; $i>=0; $i--) {
foreach ($parent\_included\_iris as $parent\_included\_iri => $parent\_included\_label) {
if (strpos($lines[$i], '=0; $i--) {
if (isset($lines[$i]) && strpos($lines[$i], '=0; $i--) {
if (isset($lines[$i]) && strpos($lines[$i], 'disjointWith')!==false) {
unset($lines[$i]);
}
}
//drop disjointWith to avoid importing too many terms
for($i=$num\_lines-1; $i>=0; $i--) {
if (isset($lines[$i]) && strpos($lines[$i], 'hasDbXref')!==false) {
unset($lines[$i]);
}
}
//drop owl:Thing
for($i=$num\_lines-1; $i>=0; $i--) {
if (isset($lines[$i]) && strpos($lines[$i], '')!==false) {
unset($lines[$i]);
}
}
for($i=$num\_lines-1; $i>=0; $i--) {
if (isset($lines[$i]) && preg\_match('/\w+:(\w+?) xmlns:\w+="([^"]+?)"/', $lines[$i], $match)){
$tmp\_iri=$match[2].$match[1];
if (!isset($processed\_iris[$tmp\_iri]) && !isset($included\_iris[$tmp\_iri]) && !iriImported($tmp\_iri)) {
$tmp\_results[$tmp\_iri] = 'NA';
}
}
}
//fix for "n0pred" beening used for diffrent xmlnss.
for($i=$num\_lines-1; $i>=0; $i--) {
if (isset($lines[$i]) && strpos($lines[$i], '");
$strOutput .= "\n$output";
preg\_match\_all('/nodeID="(\S+)"/', $output, $matches);
foreach ($matches[1] as $match) {
if (!isset($processed\_iris['nodeID://'.$match])) {
$tmp\_results['nodeID://'.$match] = 'NA';
}
}
preg\_match\_all('/resource="(.+?)"/', $output, $matches);
foreach ($matches[1] as $match) {
if (!isset($processed\_iris[$match]) && !isset($included\_iris[$match]) && !iriImported($match)) {
$tmp\_results[$match] = 'NA';
}
}
preg\_match\_all('/<\/rdf:Description>/', $output, $matches);
foreach ($matches[1] as $match) {
$strOutput .= " 


";
}
preg\_match\_all('/<\/rdf:Description>/', $output, $matches);
foreach ($matches[1] as $match) {
$strOutput .= " 


";
}
preg\_match\_all('/<\/rdf:Description>/', $output, $matches);
foreach ($matches[1] as $match) {
$strOutput .= " 


";
}
}
}
$unprocessed\_iris = $tmp\_results;
}
$strOutput .= '
';
foreach ($outputNSs as $NSTmp => $prefixTmp) {
$strOutput = "
xmlns:$prefixTmp=\"$NSTmp\"" . $strOutput;
}
$strOutput = 'xml version="1.0" encoding="utf-8" ?


Error: =$vali-getErrorMsg()?>

}
}
if ($vali-getErrorMsg()=='') {
$json\_settings = array();
$json\_settings['inputFiles'] = array();
foreach ($fileNames as $fileName=>$tmpv) {
$json\_settings['inputFiles'][]="$userfiles/$fileName.out.owl";
}
$json\_settings['outputFile'] = "$userfiles/$finalFile.owl";
$json\_settings['ontologyURI'] = $outputURI=='' ? "http://ontofox.hegroup.org/$finalFile.owl" : $outputURI;
file\_put\_contents("$userfiles/$finalFile.settings", json\_encode($json\_settings));
system("java -cp .:./OWL.jar org.hegroup.OWLMerge $userfiles/$finalFile.settings");
$fileNames[$finalFile]=1;
?>

**Finished retrieving process. Please download the output file.**

Your input file is located at http://ontofox.hegroup.org/userfiles/=$finalFile?.txt and your output file is located at http://ontofox.hegroup.org/userfiles/=$finalFile?.owl. Please includes these two links in your email if you need our assistance.

These files will be destroyed at 3:00 AM EST (New York time). If you wish to destroy these files now, please click here.

OntoFox Survey: your feedback on OntoFox is welcome and important for us to improvie this service. This survey contains 16 questions and will take approximately 5 minutes. Thank you!

$strSql="UPDATE counter SET count=count+1 WHERE page='getExternal.php'";
$db = ADONewConnection($driver);
$db-Connect($host, $username, $password, $database);
$db->Execute($strSql);
}
?>

|  |  |
| --- | --- |
| He Group  University of Michigan Medical School  Ann Arbor, MI 48109 |  |
