# Supplementary material for: OntoFox: web-based support for ontology reuse
Source: BMC Res Notes. 2010 Jun 22;3:175. doi: 10.1186/1756-0500-3-175 (PMC2911465; doi:10.1186/1756-0500-3-175)
Supplement: Additional file 3 — The source code of the OntoFox software. This zip file includes PHP source code of the OntoFox website and the Java source code of for reformatting/trimming owl (RDF/XML) output file. [file 1756-0500-3-175-S3.ZIP › website/inc/recaptcha-php-1.9/example-captcha.php]

php
require\_once('recaptchalib.php');
// Get a key from http://recaptcha.net/api/getkey
$publickey = "";
$privatekey = "";
# the response from reCAPTCHA
$resp = null;
# the error code from reCAPTCHA, if any
$error = null;
# was there a reCAPTCHA response?
if ($\_POST["recaptcha\_response\_field"]) {
$resp = recaptcha\_check\_answer ($privatekey,
$\_SERVER["REMOTE\_ADDR"],
$\_POST["recaptcha\_challenge\_field"],
$\_POST["recaptcha\_response\_field"]);
if ($resp-is\_valid) {
echo "You got it!";
} else {
# set the error code so that we can display it
$error = $resp->error;
}
}
echo recaptcha\_get\_html($publickey, $error);
?>
  
