# Supplementary material for: OntoFox: web-based support for ontology reuse
Source: BMC Res Notes. 2010 Jun 22;3:175. doi: 10.1186/1756-0500-3-175 (PMC2911465; doi:10.1186/1756-0500-3-175)
Supplement: Additional file 3 — The source code of the OntoFox software. This zip file includes PHP source code of the OntoFox website and the Java source code of for reformatting/trimming owl (RDF/XML) output file. [file 1756-0500-3-175-S3.ZIP › website/inc/recaptcha-php-1.9/example-mailhide.php]

require\_once ("recaptchalib.php");
// get a key at http://mailhide.recaptcha.net/apikey
$mailhide\_pubkey = '';
$mailhide\_privkey = '';
?
The Mailhide version of example@example.com is
 echo recaptcha\_mailhide\_html ($mailhide\_pubkey, $mailhide\_privkey, "example@example.com"); ?.   
The url for the email is:
 echo recaptcha\_mailhide\_url ($mailhide\_pubkey, $mailhide\_privkey, "example@example.com"); ?   
