# Supplementary material for: OntoFox: web-based support for ontology reuse
Source: BMC Res Notes. 2010 Jun 22;3:175. doi: 10.1186/1756-0500-3-175 (PMC2911465; doi:10.1186/1756-0500-3-175)
Supplement: Additional file 3 — The source code of the OntoFox software. This zip file includes PHP source code of the OntoFox website and the Java source code of for reformatting/trimming owl (RDF/XML) output file. [file 1756-0500-3-175-S3.ZIP › website/index.php]

OntoFox


HomeIntroductionTutorialFAQsReferencesLinksContactAcknowledge

|  |
| --- |
| **OntoFox** is a web-based system that allows users to input terms, fetch selected properties, annotations, and certain classes of related terms from the source ontologies and save the results using the RDF/XML serialization of the Web Ontology Language (OWL). OntoFox follows and expands the MIREOT principle. OntoFox also supports ontology module extratractions. OntoFox is implemented using one of the following three methods, based on how data is input and whether the OntoFox web interface is used:  1. Data input using web forms:   Examples: Example 1, example 2, example 3, example 4, example 5 **(1) Select one ontology:** |
| Please select an ontology Chemical Entities of Biological Interest (CHEBI) Common Anatomy Reference Ontology(CARO) Cell Type Ontology (CL) Human Disease Ontology (DOID) Environment Ontology (ENVO) Foundational Model of Anatomy (FMA) Gene Ontology (GO) Infectious Disease Ontology (IDO) Mammalian Phenotype Ontology (MP) NCBI Taxononmy (NCBITaxon) Ontology for Biomedical Investigations (OBI) Phenotypic Quality Ontology (PATO) Protein Ontology (PRO) Sequence Ontology (SO) Vaccine Ontology (VO) |
| **Or enter your favorite source ontology and SPARQL endpoint:** Example |
|  |
| **(2) Class term specification:** |
| **Section A: Bottom up term specification** |
| |  | | --- | | **(a) Include low level source term URIs (One URI per line):**   Search a term:  Term ID: | |  | | **(b) Include top level source term URIs and target direct superclass URIs (One URI per line, optional):**    Search a term:  Term ID: | |  | | **(c)** **Select a setting for retrieving intermediate source terms:** | | includeNoIntermediates includeComputedIntermediates includeAllIntermediates | |
| **Section B: Top down brach module extraction** |
| |  | | --- | | **(a) Include top level source term URIs and target direct superclass URIs (One URI per line):**    Search a term:  Term ID: | |  | |
| **(3) Annotation Specification: Include source annotation URIs (One URI per line, optional):** |
|  |
|  |

|  |  |
| --- | --- |
| He Group  University of Michigan Medical School  Ann Arbor, MI 48109 |  |
