# Supplementary material for: OntoFox: web-based support for ontology reuse
Source: BMC Res Notes. 2010 Jun 22;3:175. doi: 10.1186/1756-0500-3-175 (PMC2911465; doi:10.1186/1756-0500-3-175)
Supplement: Additional file 3 — The source code of the OntoFox software. This zip file includes PHP source code of the OntoFox website and the Java source code of for reformatting/trimming owl (RDF/XML) output file. [file 1756-0500-3-175-S3.ZIP › website/index_orig.php]

OntoFox


HomeIntroductionTutorialFAQsReferencesLinksContactAcknowledge

|  |
| --- |
| **OntoFox:** A web server that facilitates ontology development by automatically fetching ontology terms and their annotations from existing ontologies and saving the results in an importable RDF/OWL format. OntoFox is developed based on the MIREOT principle. OntoFox is implemented using one of the following two methods, based on how data is input:  1. Data input using web forms:   Examples: Example 1, example 2, example 3, example 4 **(1) Select one ontology:** |
| Please select an ontology Chemical Entities of Biological Interest (CHEBI) Common Anatomy Reference Ontology(CARO) Cell Type Ontology (CL) Human Disease Ontology (DOID) Environment Ontology (ENVO) Foundational Model of Anatomy (FMA) Gene Ontology (GO) Infectious Disease Ontology (IDO) Mammalian Phenotype Ontology (MP) NCBI Taxononmy (NCBITaxon) Ontology for Biomedical Investigations (OBI) Phenotypic Quality Ontology (PATO) Protein Ontology (PRO) Sequence Ontology (SO) Vaccine Ontology (VO) |
| **Or enter your favorite source ontology and SPARQL endpoint:** Example |
|  |
| **(2) Include low level source term URIs (One URI per line):**   Search a term: Term ID: |
|  |
| **(3) Include top level source term URIs and target direct superclass URIs (One URI per line, optional):**    Search a term: Term ID: |
|  |
| **(4)** **Select a setting for retrieving intermediate source terms:** |
| includeNoIntermediates includeComputedIntermediates includeAllIntermediates |
| **(5) Include source annotation URIs (One URI per line, optional):** |
|  |
|  |


|  |
| --- |
| 2. Data input using local text file: |
| **Upload input file:** |
|  |

OntoFox Survey: your feedback on OntoFox is welcome and important for us to improvie this service. This survey contains 16 questions and will take approximately 5 minutes. Thank you!

|  |  |
| --- | --- |
| He Group  University of Michigan Medical School  Ann Arbor, MI 48109 |  |
