# Supplementary material for: OntoFox: web-based support for ontology reuse
Source: BMC Res Notes. 2010 Jun 22;3:175. doi: 10.1186/1756-0500-3-175 (PMC2911465; doi:10.1186/1756-0500-3-175)
Supplement: Additional file 3 — The source code of the OntoFox software. This zip file includes PHP source code of the OntoFox website and the Java source code of for reformatting/trimming owl (RDF/XML) output file. [file 1756-0500-3-175-S3.ZIP › website/introduction.php]

OntoFox


HomeIntroductionTutorialFAQsReferencesLinksContactAcknowledge

### Introduction

Developing a biomedical ontology covering a specific domain (e.g., OBI for biomedical investigations, VO for vaccine) is often an ambitious project. OBO Foundry principles have been developed to ensure collaborative ontology development and interoperability among ontologies. Examples of these principles include: a) ontologies are developed in a collaborative effort, b) ontologies use
common relations that are unambiguously defined, c) ontologies provide procedures for user feedback and for
identifying successive versions, and d) ontologies have a clearly bounded subject-matter.

To avoid duplication of effort during ontology development, it is advised to import pre-existing ontology terms and knowledge into a new ontology if possible. The Web Ontology Language (OWL) provides a mechanism to import ontologies. This approach leads to import of the whole ontology. However, importing a whole ontology may not be practical and needed, especially when the source ontology is very large and in most cases not relevant. For example, the Ontology for Biomedical Investigations (OBI) studies investigations in different biomedical areas and uses many terms from other biomedical ontologies. It is impractical to import all these other ontologies as a whole into OBI. The development of Vaccine Ontology (VO) requires importing of a large number of NCBI taxonomy terms. It is laborious to manually import the relevant information and practically impossible to keep update the manually imported information.

To address this challenge, the OBI Consortium proposes a method to allow selective use of classes from external ontologies that are of direct interest to OBI. The principle of MIREOT (Minimum Information to Reference External Ontology Terms) is eventually developed. A set of MIREOT guidelines have also been recommended for consistent reference of an external term. The MIREOT guideline suggests the following minimal set: (1) source ontology URI; (2) source term URI; and (3) target direct superclass URI. The MIREOT guideline has been applied in an OBI MIREOT program using scripts and command lines. However, the OBI MIREOT was developed for helping OBI development and cannot be used for development of other ontologies without modification. Its usage also requires programming using SPARQL, scripts, and command lines. This restricts its wide uses among interesting ontology developers.

OntoFox is a web-based system that follows and extends the MIREOT guidelines. OntoFox requests the following data input: (1) Source ontology URI. (2) Low level source term URI (equivalent to "source term URI" in the MIREOT guideline). (3) Top level source term URI. Compared to the minimum data set from the MIREOT guideline, the top level source term URI is added in order to automatically retrieve any intermediate ontology terms between the low and top level source terms in the source ontology. (4) URI of target direct superclass of top level source term. Since each top level source term has its own direct superclass in the target ontology, this data is input together with the top level source term in OntoFox input format. (5) Source term annotation URIs. The source term annotation URIs are requested in case only limited annotations are needed. If no annotation URI is assigned, all annotations associated with a specific ontology term will be fetched.

OntoFox also develops a SPARQL-based ontology module extraction algorithm. Our algorithm allows extraction of external ontology terms relevant to a given set of signature terms, implemented by SPARQL query of associated RDF triples. Effectively the algorithm takes the union of the concise bounded description (cite http://www.w3.org/Submission/CBD/) of the terms. This algorithm extracts additional terms that are not in the is-a hierarchies of signature terms. This SPARQL modularization algorithm is execuated in OntoFox after a user selects "includeAllIntermediates” and “includeAllAxioms”. The benefit of using SPARQL is that it is highly scalable – current modularization algorithms use in-memory representations.

The output RDF/OWL file can be directly imported in the target ontology using the OWL import function. The output file can be visualized using Protege or other OWL editors. The annotation information of low level source term, top level source term, all terms in between the low and top level source terms in the ontology hierarchy, and terms and annotations that are related to the signature terms but may not in the is\_a hierarchies will all be fetched by OntoFox.

Since source ontologies may be under active development and updates, it is advised that the OntoFox process is implemented periodically to import most up-to-date information of external ontology terms.

With more ontologies being developed, OntoFox offers a timely web-based package of solutions for ontology imports via MIREOT and ontology module extraction. OntoFox provides an efficient approach to promote ontology sharing and interoperability. It is easy to use and does not require knowledge of SPARQL, script programming, and command line operation. OntoFox is developed to serve the ontology community for ontology reuse. It is freely available under the Apache License 2.0.

Suggestions and comments are welcome. Thank you!

|  |  |
| --- | --- |
| He Group  University of Michigan Medical School  Ann Arbor, MI 48109 |  |
