# Supplementary material for: OntoFox: web-based support for ontology reuse
Source: BMC Res Notes. 2010 Jun 22;3:175. doi: 10.1186/1756-0500-3-175 (PMC2911465; doi:10.1186/1756-0500-3-175)
Supplement: Additional file 3 — The source code of the OntoFox software. This zip file includes PHP source code of the OntoFox website and the Java source code of for reformatting/trimming owl (RDF/XML) output file. [file 1756-0500-3-175-S3.ZIP › website/links.php]

OntoFox


HomeIntroductionTutorialFAQsReferencesLinksContactAcknowledge

### Links

**Biomedical Ontologies:**

Ontologies in OBO Foundry: http://www.obofoundry.org

CARO: Common Anatomy Reference Ontology: http://www.obofoundry.org/cgi-bin/detail.cgi?id=caro

CHEBI: Chemical Entities of Biological Interest: http://chebi.wiki.sourceforge.net/

CL: Cell Type Ontology: http://www.obofoundry.org/cgi-bin/detail.cgi?id=cell

DOID: Human Disease Ontology: http://diseaseontology.sourceforge.net/

ENVO: Environment Ontology: http://environmentontology.org/

GO: Gene Ontology: http://www.geneontology.org

IDO: Infectious Disease Ontology: http://www.bioontology.org/wiki/index.php/Infectious\_Disease\_Ontology

NCBITaxon: NCBI Taxonomy: http://www.ncbi.nlm.nih.gov/Taxonomy/

MP: Mammalian Phenotype Ontology: http://www.informatics.jax.org/searches/MP\_form.shtml

OBI: Ontology for Biomedical Investigations: http://obi-ontology.org

PATO: Phenotypic Quality Ontology: http://www.obofoundry.org/cgi-bin/detail.cgi?id=quality

PRO: Protein Ontology: http://www.obofoundry.org/cgi-bin/detail.cgi?id=protein

SO: Sequence Ontology: http://www.sequenceontology.org/

VO: Vaccine Ontology: http://www.violinet.org/vaccineontology

**Tools:**

Protege ontology editor: http://protege.stanford.edu/

NCBO BioPortal: http://bioportal.bioontology.org/

VO Browser: http://www.violinet.org/vaccineontology/vobrowser/ (Note: VO uses OntoFox for its own development. For example, ~400 NCBITaxon and ~300 MP terms and their annotations have been imported into VO using OntoFox.)

|  |  |
| --- | --- |
| He Group  University of Michigan Medical School  Ann Arbor, MI 48109 |  |
