# Supplementary material for: OntoFox: web-based support for ontology reuse
Source: BMC Res Notes. 2010 Jun 22;3:175. doi: 10.1186/1756-0500-3-175 (PMC2911465; doi:10.1186/1756-0500-3-175)
Supplement: Additional file 3 — The source code of the OntoFox software. This zip file includes PHP source code of the OntoFox website and the Java source code of for reformatting/trimming owl (RDF/XML) output file. [file 1756-0500-3-175-S3.ZIP › website/references.php]

OntoFox


HomeIntroductionTutorialFAQsReferencesLinksContactAcknowledge

### References

Courtot M, Gibson F, Lister AL, Malone J, Schober D, Brinkman RR, Ruttenberg A. MIREOT: the Minimum Information to Reference an External Ontology Term. ICBO 2009.

MIREOT (Minimal information to reference external ontology terms): http://obi-ontology.org/page/MIREOT.

Ruttenberg A, Rees JA, Samwald M, Marshall MS. Life sciences on the Semantic Web: the Neurocommons and beyond. *Brief Bioinform*. 2009 Mar;10(2):193-204. PMID: 19282504.

Smith B, Ashburner M, Rosse C, Bard J, Bug W, Ceusters W, Goldberg LJ, Eilbeck K, Ireland A, Mungall CJ; OBI Consortium, Leontis N, Rocca-Serra P, Ruttenberg A, Sansone SA, Scheuermann RH, Shah N, Whetzel PL, Lewis S. (2007). The OBO Foundry: coordinated evolution of ontologies to support biomedical data integration. *Nat Biotechnol* 25 (11): 1251-5. PMID 17989687.

|  |  |
| --- | --- |
| He Group  University of Michigan Medical School  Ann Arbor, MI 48109 |  |
