# Supplementary material for: OntoFox: web-based support for ontology reuse
Source: BMC Res Notes. 2010 Jun 22;3:175. doi: 10.1186/1756-0500-3-175 (PMC2911465; doi:10.1186/1756-0500-3-175)
Supplement: Additional file 3 — The source code of the OntoFox software. This zip file includes PHP source code of the OntoFox website and the Java source code of for reformatting/trimming owl (RDF/XML) output file. [file 1756-0500-3-175-S3.ZIP › website/remove.php]

OntoFox


HomeIntroductionTutorialFAQsReferencesLinksContactAcknowledge

include('inc/functions.php');
$vali=new Validation($\_REQUEST);
$f= $vali-getInput('f', 'F', 0, 128);
if ($f!='') {
$tokens = preg\_split('/,/', $f);
foreach ($tokens as $token) {
if (preg\_match('/^[a-z0-9]+$/', $token)) {
system ("rm -f userfiles/$token.\*");
}
}
}
?>

Files have been destroyed!

|  |  |
| --- | --- |
| He Group  University of Michigan Medical School  Ann Arbor, MI 48109 |  |
