# Supplementary material for: OntoFox: web-based support for ontology reuse
Source: BMC Res Notes. 2010 Jun 22;3:175. doi: 10.1186/1756-0500-3-175 (PMC2911465; doi:10.1186/1756-0500-3-175)
Supplement: Additional file 3 — The source code of the OntoFox software. This zip file includes PHP source code of the OntoFox website and the Java source code of for reformatting/trimming owl (RDF/XML) output file. [file 1756-0500-3-175-S3.ZIP › website/tutorial/index.php]

OntoFox


HomeIntroductionTutorialFAQsReferencesLinksContactAcknowledge

### Tutorial

Here we provide a tutorial of how OntoFox can be applied for your research and ontology development:

Table of Contents

1. Source Ontologies and their namespaces
2. OntoFox execution using web input forms
3. OntoFox data input format
4. Four "directives" used in OntoFox
5. Four settings used in OntoFox
6. OntoFox hands on demo
7. OntoFox use case demostrations
8. OntoFox access without using OntoFox web site

1. Source Ontologies and their namespaces:   
OntoFox currently includes the following source ontologies for query. OntoFox uses standardized namespaces of source ontologies following OBO Foundry recommendations. These namespaces are also used in ontology servers like Neurocommons.org:

> | # | Ontology | Base URI | Term URI example |
> | --- | --- | --- | --- |
> | **1** | CARO | http://purl.org/obo/owl/CARO | http://purl.org/obo/owl/CARO#CARO\_0000040 |
> | **2** | **CHEBI** | http://purl.org/obo/owl/CHEBI | http://purl.org/obo/owl/CHEBI#CHEBI\_48999 |
> | **3** | CL | http://purl.org/obo/owl/CL | http://purl.org/obo/owl/CL#CL\_0000799 |
> | **4** | DOID | http://purl.org/obo/owl/DOID | http://purl.org/obo/owl/DOID#DOID\_12685 |
> | **5** | ENVO | http://purl.org/obo/owl/ENVO | http://purl.org/obo/owl/ENVO#ENVO\_00000483 |
> | **6** | FMA | http://purl.org/obo/owl/FMA | http://purl.org/obo/owl/FMA#FMA\_9712 |
> | **7** | GO | http://purl.org/obo/owl/GO | http://purl.org/obo/owl/GO#GO\_0043152 |
> | **8** | IDO | http://purl.obolibrary.org/obo/ | http://purl.obolibrary.org/obo/IDO\_0000064 |
> | **9** | MP | http://purl.org/obo/owl/MP | http://purl.org/obo/owl/MP#MP\_0000026 |
> | **10** | **NCBITaxon** | http://purl.org/obo/owl/NCBITaxon | http://purl.org/obo/owl/NCBITaxon#NCBITaxon\_263 |
> | **11** | **OBI** | http://purl.obolibrary.org/obo/ | http://purl.obolibrary.org/obo/OBI\_0100026 |
> | **12** | PATO | http://purl.org/obo/owl/PATO | http://purl.org/obo/owl/PATO#PATO\_0001793 |
> | **13** | PRO | http://purl.org/obo/owl/PRO | http://purl.org/obo/owl/PRO#PRO\_000001795 |
> | **14** | SO | http://purl.org/obo/owl/SO | http://purl.org/obo/owl/SO#SO\_0001288 |
> | **15** | VO | http://purl.obolibrary.org/obo/ | http://purl.obolibrary.org/obo/VO\_0000001 |

2. OntoFox execution using web input forms:   
Data for each component can be input using the web input form in the OntoFox home page. OntoFox needs the following information as input from users:

**(1) Source ontology:** The ontology where a list of terms will be retrieved from.

**(2) Low level source term URIs****:** The URIs of low level term from source ontologies.

**(3) Top level source term URIs and **tTarget direct superclass URIs******:** The URIs of top level term from source ontologies and their direct superclass URIs from target ontologies. The top level source term URI can be the same as the low level source term URI. In this case, a single source term will be fetched. If no top level source term is specified, by default the top level source term is the same as the low level source term. Since each top level source term has its own superclass in the target ontology, each target direct superclass of a top level source term should be specified. In OntoFecth, we specify the target direct superclass in a new line, following the sign "subClassOf ".

**(4) Setting for retrieving intermediate source terms:**  Three options are available for retrieving intermediate terms: (a) **includeNoIntermediates**: no intermediate source terms are retrieved. (b) **includeComputedIntermediates**: Sensible intermediate source terms are retrieved. Sensible intermediates include those intermediate terms that are closest ancestors of more than one low level source terms. (c) **includeAllIntermediates**: All intermediate source terms are retrieved.

**(5) Source annotation URIs:** The annotation URIs for the source terms used in the source ontologies. To map or wrap an annotation to a new one, please specify "copyTo" or "mapTo" in the new line following the orignal annotation URL, followed by the new annotation URI.

The OntoFox home page provide many examples. These examples can be used to quickly learn how to use the OntoFox web forms for OntoFox implementation.

Common annotation URIs:

|  |  |
| --- | --- |
| rdfs:label | http://www.w3.org/2000/01/rdf-schema#label |
| oboInOwl:hasSynonym | http://www.geneontology.org/formats/oboInOwl#hasSynonym |
| oboInOwl:hasExactSynonym | http://www.geneontology.org/formats/oboInOwl#hasExactSynonym |
| oboInOwl:hasRelatedSynonym | http://www.geneontology.org/formats/oboInOwl#hasRelatedSynonym |
| oboInOwl:hasNarrowSynonym | http://www.geneontology.org/formats/oboInOwl#hasNarrowSynonym |
| oboInOwl:hasBroadSynonym | http://www.geneontology.org/formats/oboInOwl#hasBroadSynonym |
| oboInOwl:hasDefinition | http://www.geneontology.org/formats/oboInOwl#hasDefinition |
| iao:preferredTerm | http://purl.obolibrary.org/obo/IAO\_0000111 |
| iao:definition | http://purl.obolibrary.org/obo/IAO\_0000115 |
| iao:alternative term | http://purl.obolibrary.org/obo/IAO\_0000118 |

  

3. OntoFox data input format:    
An example of OntoFox data input file is here:

> -----------------------
>
> [Source ontology]  
> #comment here  
> #List of ontologies: OBI, NCBITaxon, MP, PATO, GO, DOID, IDO, CHEBI, SO, PRO, CL, ENVO, CARO   
> NCBITaxon  
> OBI #another comment here
>
> [Low level source term URIs]  
> http://purl.org/obo/owl/NCBITaxon#NCBITaxon\_263 #Francisella tularensis   
> http://purl.org/obo/owl/NCBITaxon#NCBITaxon\_234 #Brucella  
> http://purl.org/obo/owl/PATO#PATO\_0001793 #right side of   
> http://purl.org/obo/owl/PATO#PATO\_0001792 #left side of
>
> [Top level source term URIs and target direct superclass URIs]  
> http://purl.org/obo/owl/NCBITaxon#NCBITaxon\_2 #Bacteria   
> subClassOf http://purl.obolibrary.org/obo/OBI\_0100026 #organism, this term is from target ontology   
> http://purl.org/obo/owl/PATO#PATO\_0001238   
> subClassOf http://www.ifomis.org/bfo/1.1/snap#Quality #Note: Use designated sign "subClassOf"
>
> [Source term retrieval setting]  
> includeNoIntermediates #or: includeAllIntermediates, inincludeComputedIntermediates
>
> [Source annotation URIs]  
> http://www.w3.org/2000/01/rdf-schema#label  
> copyTo http://purl.obolibrary.org/obo/IAO\_0000111  
> http://www.geneontology.org/formats/oboInOwl#hasDefinition  
> mapTo http://purl.obolibrary.org/obo/IAO\_0000115  
> http://www.geneontology.org/formats/oboInOwl#hasSynonym  
>   
> -----------------------

As you can tell, the OntoFox data input format contains the following components:

1. **Headings:** Each heading contains a text description quoted inside parenthesis "[ ]". Five headings represent the four components of OntoFox execution. Please use the exact same text in all headings.
2. **Source ontology list under the first heading.** This is required. If using web forms, one or many ontologies can be selected. Currently, the ontologies we have tested and included in OntoFox include: OBI, NCBITaxon, MP, PATO, GO, DOID, IDO, CHEBI, SO, PRO, CL, ENVO, and CARO.
3. **Low level source term URIs:** At least one source term URI is required.
4. **Top level source term URIs and target direct superclass URIs:** Since each top level source term has its own direct superclass in the target ontology, URIs of target direct superclasses of individual top level source terms are input together with the top level source term with a new line starting with "**subClassOf**". To get a single class from source ontology, you do not need to specify any top level source term, or you can specify the top level source term URI as the same as the low level source term URI.
5. **Source term retrieval setting:** Choose one of three settings for retrieving intermediate source ontology terms:: includeNoIntermediates, includeAllIntermediates, and inincludeComputedIntermediates. See description below.
6. **Source annotation URIs:** The source term annotation URIs are requested in case only limited annotations are needed. If no annotation URI is assigned, no annotations associated with a specific ontology term will be fetched. To include all possible annotations, you can put "**includeAllAxioms**"on one line, and all the annotations associated with a specific ontology term will be fetched. To map or wrap an annotation to a new one, please specify "copyTo" or "mapTo" in the new line following the orignal annotation URL, followed by the new annotation URI.
7. **Comments:** The sign "#" as the first letter of a line or as a letter after a space in the middle of a line is an indicator of a comment. All text after this sign within one line is considered comment and will not be used for OntoFox analysis.

|  |  |
| --- | --- |
| He Group  University of Michigan Medical School  Ann Arbor, MI 48109 |  |
